# Evaluation of gestational age, serum amyloid A, and hemoglobin decline (ΔHb) as diagnostic markers for NEC secondary to late-onset sepsis in preterm infants

**DOI:** 10.3389/fped.2025.1662371

**Published:** 2025-10-09

**Authors:** Yun Cheng, Biquan Chen, Wenjia Tong, Wangqiang Li, Yuanyuan Duan

**Affiliations:** Department of Infection, Anhui Provincial Children’s Hospital, Hefei, China

**Keywords:** serum amyloid A, late-onset sepsis, necrotizing enterocolitis, hemoglobin, preterm infants, gestational age

## Abstract

**Objective:**

To evaluate gestational age, serum amyloid A (SAA), and hemoglobin decline (ΔHb) as diagnostic markers for necrotizing enterocolitis (NEC) secondary to late-onset sepsis (LOS) in preterm infants.

**Methods:**

A retrospective study was conducted on 77 preterm infants with LOS admitted to Anhui Provincial Children's Hospital from January 1, 2019, to October 31, 2024. The infants were divided into an NEC group (24 cases) and a non-NEC group (53 cases). Perinatal factors, initial blood counts, C-reactive protein, and SAA levels during early LOS were recorded. ΔHb was calculated as the difference between pre-LOS Hb concentration and initial Hb concentration at LOS onset. Differences were analyzed using the Mann–Whitney *U* test, *χ*^2^ test, or Fisher's exact test. ROC curves were used to evaluate the predictive value.

**Results:**

The NEC group had significantly lower birth weight, gestational age, white blood cell count, neutrophil count, and lymphocyte count compared to the non-NEC group. In contrast, SAA, ΔHb, asphyxia incidence, and ventilator use rate were significantly higher in the NEC group (*P* < 0.05). Logistic regression analysis indicated that gestational age, SAA, and ΔHb were independent risk factors for NEC secondary to LOS. ROC curve analysis showed that the optimal cut-off values were 236 days for gestational age, 78.3 mg/L for SAA, and 15 g/L for ΔHb. The combined model had an area under the curve of 0.888 (95% CI: 0.810–0.953, *P* < 0.001), and the Youden's index was 0.637.

**Conclusion:**

Gestational age, SAA, and ΔHb appear to be useful indicators for predicting NEC secondary to LOS in preterm infants. The ease of obtaining these indicators and their low cost make them suitable for clinical application. However, this study was retrospective with certain uncontrollable factors and a relatively small sample size. Larger prospective studies are recommended for further validation.

## Introduction

Neonatal necrotizing enterocolitis (NEC) is an intestinal disease with an unclear mechanism, characterized by a sudden onset and rapid progression. It is common in preterm and low birth weight infants and is one of the primary causes of neonatal mortality, with a fatality rate of up to 20%–30%. Complications such as intestinal stricture, short bowel syndrome, and neurological sequelae contribute to poor neonatal outcomes (1, 2).

Studies have shown that infection plays a significant role in the development of NEC (3, 4), with 34%–57% of sepsis cases leading to NEC (5). Therefore, identifying effective early predictors for NEC in preterm infants with sepsis is crucial for reducing NEC incidence and mortality. Serum amyloid A (SAA) is an acute-phase protein synthesized by hepatocytes in response to infection, trauma, or ischemic injury and is a biomarker for various diseases (6–8). Elevated SAA levels are observed early in neonatal sepsis and NEC, reflecting disease severity and treatment response, making it a useful nonspecific marker for early diagnosis of bacterial infections like sepsis and NEC.

Approximately 60% of infants with sepsis also develop anemia (9), and anemia has been shown to be a risk factor for NEC (10, 11).Based on the established pathophysiological links between inflammation, anemia of inflammation, and gut injury in prematurity, we hypothesized that the combination of immaturity (as reflected by lower gestational age), a pronounced acute-phase inflammatory response (as measured by SAA), and a rapid drop in hemoglobin (ΔHb, indicative of anemia of inflammation) would together provide a robust early signal identifying preterm infants with late-onset sepsis (LOS) who are at highest risk for progressing to NEC.

Therefore, this study was designed to specifically and prospectively evaluate this predefined set of biomarkers within the high-risk context of LOS. Studies investigating gestational age, SAA, and ΔHb in relation to NEC secondary to LOS in preterm infants were limited. This study aimed to explore the relationship between these indicators and NEC in preterm infants with LOS, to provide potentially accessible and reliable biomarkers.

## Methods

### Study design and participants

A retrospective analysis was conducted on 77 preterm infants with LOS admitted to Anhui Provincial Children's Hospital from January 1, 2019, to October 31, 2024. The infants included 58 males and 19 females, and they were divided into an NEC group (24 cases) and a non-NEC group (53 cases) based on whether NEC occurred post-LOS. This study complied with the Declaration of Helsinki and was approved by the Ethics Committee of Anhui Provincial Children's Hospital. Due to the retrospective nature of the study, the committee waived the informed consent form.

### Diagnostic criteria

The diagnosis of late-onset neonatal sepsis was based on the criteria by Haque et al (12), including clinical and laboratory variables. Confirmed cases had positive blood cultures and clinical signs of infection. Suspected sepsis cases had clinical signs but lacked pathogen identification, potentially due to low blood culture sensitivity, with significant laboratory changes (at least two altered results). Cases with elevated CRP (>10 mg/L) but lacking definitive signs were classified as probable sepsis (12). NEC was diagnosed using the modified Bell's criteria, which include clinical symptoms (e.g., bile-stained gastric aspirate, vomiting, abdominal distension, and visible or occult blood in stools without anal fissures) and one or more radiographic or ultrasound signs (e.g., pneumatosis intestinalis, portal venous gas, pneumoperitoneum) (13). Based on the severity of the illness and in reference to the neonatal shock score, LOS (late-onset sepsis) was classified as severe LOS (characterized by a critical clinical condition, with shock and a shock score >6) and mild LOS (characterized by a milder clinical condition, without shock or with shock but a shock score ≤6) (14).

### Inclusion and exclusion criteria

Inclusion criteria: (1) Preterm infants with gestational age <37 weeks; (2) Meeting neonatal LOS diagnostic criteria (12).

Exclusion criteria: (1) Early-onset sepsis (age ≤3 days); (2) Meconium aspiration syndrome; (3) Genetic metabolic disorders, congenital anomalies, or hematological diseases; (4) Hb decline due to bleeding conditions or iatrogenic blood loss within the testing interval; (5) Transfusion within 72 h before the onset of NEC-related sympto.; (6) Symptoms related to NEC appeared within 72 h of sepsis; (7) those who received red blood cell transfusions before the occurrence of LOS.

### Definition of NEC secondary to LOS and timing

For the purpose of this study, we defined a case of “NEC secondary to LOS” as a diagnosis of NEC (modified Bell's stage ≥IIA) that occurred more than 72 h after the clinical onset of LOS. This temporal criterion was established to ensure a clear sequence of events where LOS unequivocally preceded the development of NEC, thereby reducing the likelihood of misclassifying co-incident conditions.

The onset of LOS was defined as the time when the first clinical signs (e.g., fever, temperature instability, apnea, or poor responsiveness) were documented in the medical record, which prompted the clinical team to initiate a sepsis evaluation and draw the first blood culture.

### Handling of NEC signs near LOS onset

Infants who exhibited signs of NEC (e.g., abdominal distension, bloody stools) within the first 72 h after LOS onset were carefully evaluated. These cases were excluded from the “NEC secondary to LOS” group if the NEC diagnosis was confirmed within this window. This was to avoid inclusion of cases where NEC might have been incubating concurrently with or prior to the clinical presentation of LOS, ensuring a clearer causal temporal relationship for our analysis.

### Selection of laboratory tests

**Figure 1:**
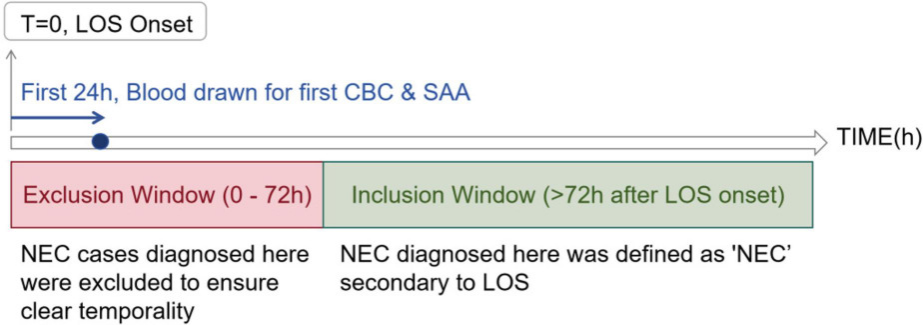
Flowchart of the study timeline defining late-onset sepsis (LOS) onset and secondary necrotizing enterocolitis (NEC).

For infants with multiple blood tests within the first 24 h of LOS onset, we used the results from the first available complete blood count (CBC) and serum amyloid A (SAA) sample obtained after the documented clinical onset. This approach was chosen to best capture the earliest hematological and inflammatory response at the time of clinical suspicion, which is most relevant for early risk stratification prior to the availability of culture results (Figure 1).

### Blood test selection

Timeline showing the onset of late-onset sepsis (LOS) at time zero. The first 24 hours involve drawing blood for the first complete blood count (CBC) and serum amyloid A (SAA). An exclusion window from 0 to 72 hours removes NEC cases to ensure clear temporality, while the inclusion window starts after 72 hours. NEC diagnosis here is considered secondary to LOS.

### Data collection

We collected clinical data of the children, including gestational age, birth weight, sex, age at onset, perinatal conditions, and the use of red blood cell transfusions and ventilators after the occurrence of LOS and before the occurrence of NEC. We recorded the most recent complete blood count (CBC) results prior to the occurrence of LOS. We defined the most recent CBC results prior to the occurrence of LOS as normal and the child as having no clinical infection symptoms as “before the occurrence of LOS.” We defined the clinical manifestations of LOS, such as fever and poor responsiveness, without NEC symptoms as “early stage of LOS.” We calculated ΔHb as the difference between the most recent Hb level prior to the occurrence of LOS and the Hb level at the early stage of LOS. The timing of blood draws for hemoglobin measurement was recorded to the nearest calendar day. The exact time of day was not consistently available in the medical records. Therefore, the interval between paired measurements is reported in whole days, and the calculation of a precise rate of change (e.g., ΔHb per day) was not feasible.

### Statistical analysis

Data were analyzed using SPSS 20.0 and R software. Continuous data were expressed as mean ± standard deviation for normally distributed variables, compared using independent *t*-tests, or as median (interquartile range) for non-normal distributions, compared using Mann–Whitney *U* or Kruskal–Wallis *H* tests. Categorical data were expressed as frequencies and percentages, with group comparisons conducted using *χ*^2^ or Fisher's exact test. Due to the limited number of events (NEC cases = 24), we avoided automated variable selection procedures (e.g., stepwise regression) to prevent overfitting and model instability. Instead, a multivariable logistic regression model was pre-specified based on clinical-pathophysiological rationale to include our primary variables of interest: gestational age (GA), serum amyloid A (SAA), and hemoglobin decline (ΔHb). In case of a strong correlation (defined as *ρ* > 0.7), a preemptive clinical decision was made to include only the most etiologically fundamental variable in the final multivariate model to ensure model parsimony and avoid inflating the variance of the coefficient estimates. ROC curves were constructed to determine cut-off values, sensitivity, and specificity for these predictive factors. To account for the limited sample size and potential overoptimism of the predictive model, internal validation was performed using bootstrap resampling with 1,000 repetitions to calculate an optimism-corrected area under the curve (AUC).

### Variable selection and primary model specification

Outcome. The primary outcome was NEC secondary to LOS, defined as NEC (Bell's stage ≥IIA) diagnosed more than 72 h after the clinical onset of LOS (*T* = 0).

Candidate predictors. Gestational age (GA, days), serum amyloid A (SAA, mg/L), and hemoglobin decline (ΔHb, g/L). Penalization & scaling. Predictors were standardized (*z*-scores), and L1-penalized logistic regression (LASSO) was applied.

Cross-validation & λ selection. Ten-fold cross-validation was used with AUROC as the performance metric. The penalty parameter was selected at λ_min (the value achieving maximal AUROC). Software. Analyses were performed using Python 3.11.8, scikit-learn 1.1.3, statsmodels 0.13.5, numpy 1.24.0, and pandas 1.5.3.

Role of LASSO. LASSO was used solely for variable selection. All three predictors were retained, and a standard (unpenalized) logistic regression was refit with GA, SAA, and ΔHb as the primary model for reporting and clinical application.

## Results

### Assessment of multicollinearity

As anticipated, a very strong positive correlation was observed between gestational age and birth weight (Spearman's *ρ* = 0.828, *p* < 0.001). This justified our pre-specified model building strategy to include only gestational age in the subsequent multivariate logistic regression analysis to avoid multicollinearity.

### Comparison of clinical data between NEC and non-NEC groups

As shown in Tables 1, 2, no significant differences were observed between the NEC and non-NEC groups in gender, age, age of onset, shock scores, disease severity, platelet count, CRP, mean platelet volume, red cell distribution width, and Hb levels pre- and post-LOS. However, the NEC group had significantly lower birth weight, gestational age, white blood cell count, neutrophil count, and lymphocyte count, with higher SAA levels, ΔHb, asphyxia incidence, and ventilator usage rate compared to the non-NEC group (*P* < 0.05).

**Table 1:** Comparison of the general situation of LOS children in the two groups.

Variables		Non-NEC (*n* = 53)	NEC (*n* = 24)	*χ*2	*p*
Gender	Male	39 (73.58)	19 (79.17)	0.277	0.599
	Female	14 (26.42)	5 (20.83)		
LOS	Mild	45 (84.91)	23 (95.83)	1.911	0.167
	Severe	8 (15.09)	1 (4.17)		
Primipara	Yes	23 (43.40)	10 (41.67)	0.02	0.887
	No	30 (56.60)	14 (58.33)		
Cesarean section	Yes	27 (50.94)	10 (41.67)	0.57	0.45
	No	26 (49.06)	14 (58.33)		
Polyembryony	Yes	15 (28.30)	10 (41.67)	1.346	0.246
	No	38 (71.70)	14 (58.33)		
Breast-feeding	Yes	24 (45.28)	9 (37.50)	0.409	0.523
	No	29 (54.72)	15 (62.50)		
Premature rupture of membranes >18 h	No	47 (88.68)	22 (91.67)	0.158	0.691
	Yes	6 (11.32)	2 (8.33)		
Maternal gestational diabetes	No	46 (88.46)	21 (87.50)	0.015	0.904
	Yes	6 (11.54)	3 (12.50)		
Maternal hypertension in pregnancy	No	46 (86.79)	21 (87.50)	0.007	0.932
	Yes	7 (13.21)	3 (12.50)		
Asphyxia	No	31 (58.49)	6 (25.00)	7.423	0.006
	Yes	22 (41.51)	18 (75.00)		
Ventilator usage	No	34 (64.15)	8 (33.33)	6.328	0.012
	Yes	19 (35.85)	16 (66.67)		
PDA	No	47 (88.68)	21 (87.50)	0.022	0.881
	Yes	6(11.32)	3(12.50)		

Data are presented as *n* (%). LOS, late-onset sepsis; PROM, premature rupture of membranes.

PDA, patent ductus arteriosus.

**Table 2:** Comparison of initial LOS laboratory test results and hemoglobin decline values between the two groups.

Variables	Non-NEC (*n* = 53)	NEC (*n* = 24)	z/t	*p*
age [M(P25, P75), d]	15.0 (9.0, 20.0)	14.5 (7.3, 20.0)	−0.099	0.921
shock scores [M(P25, P75)]	9.0 (8.0, 10.0)	9.0 (8.3, 10.0)	−0.204	0.838
GA [M(P25, P75), d]	241.0 (216.0, 252.0)	219.0 (212.0, 235.0)	−2.933	0.003
BW [M(P25, P75), g]	1,950.0 (1,555.0,2, 550.0)	1,540.0 (1,303.8,1, 837.5)	−2.398	0.016
age of onset [M(P25, P75), d]	15.0 (9.0, 20.5)	14.5 (7.3, 19.8)	−0.224	0.823
WBC [M(P25, P75), ×10^9^/L]	10.6 (4.7, 21.8)	5.7 (3.1, 9.0)	−2.925	0.003
N [M(P25, P75), ×10^9^/L]	4.8 (2.8, 14.4)	2.6 (1.5, 6.6)	−2.150	0.032
L [M(P25, P75), ×10^9^/L]	2.7 (1.4, 4.3)	1.3 (0.9, 2.4)	−3.800	0.000
PLT [M(P25, P75), ×10^9^/L]	164.0 (98.0, 278.0)	176.5 (103.5, 256.5)	−0.011	0.991
Pre-los hemoglobin [(M ± SD), g/L]	121.3 ± 24.4	119.9 ± 26.6	0.234	0.815
CRP [M(P25, P75), mg/L]	26.0 (4.5, 74.7)	44.4 (21.5, 73.2)	−1.859	0.063
MPV [M(P25, P75), fl]	10.9 (10.4, 12.0)	11.2 (10.5, 11.5)	−0.16	0.873
Red cell distribution width [(M ± SD),%]	63.4 ± 9.8	64.0 ± 8.9	−0.267	0.79
Post-LOS hemoglobin [M(P25, P75), g/L]	100.0 (88.0, 118.0)	98.5 (82.8, 105.8)	−1.513	0.13
SAA [M(P25, P75), mg/L]	54.3 (18.9, 129.1)	145.8 (79.3, 181.7)	−3.124	0.002

Data are presented as M(P25, P75) [Median (25th, 75th Percentile)] or mean ± SD. GA, gestational age (days); BW, birth weight (g); LOS, late-onset sepsis; WBC, white blood cell count (×10⁹/L); N, neutrophil count (×10⁹/L); L, lymphocyte count (×10⁹/L); MPV, mean platelet volume (fL); CRP, C-reactive protein (mg/L); SAA, serum amyloid A (mg/L); ΔHb, hemoglobin decline (g/L); age, postmenstrual age (days) at admission for LOS; age of onset, postmenstrual age (days) at clinical onset of LOS.

### Logistic regression analysis for NEC secondary to LOS

Using whether NEC occurred as the dependent variable, we conducted a multifactorial logistic regression analysis with the statistically significant indicators mentioned above, including gestational age, birth weight, white blood cell count, neutrophil count, lymphocyte count, SAA, ΔHb, incidence of asphyxia, and ventilator use rate, as independent variables. As shown in Table 3, gestational age, SAA, and ΔHb were independent risk factors for the occurrence of NEC in premature infants.

**Table 3:** Multi-factor analysis of LOS secondary NEC.

Item	Regression coefficient	Standard error	*Z* value	Wald χ2	*p*-Value	OR 95% CII
GA	−0.148	0.056	−2.633	6.933	0.008	0.862 (0.772–0.963)
BW	0.001	0.001	0.729	0.532	0.466	1.001 (0.998–1.004)
WBC	−0.719	0.733	−0.981	0.962	0.327	0.487 (0.116––2.050)
N	0.647	0.749	0.863	0.745	0.388	1.91 (0.440–8.296)
L	−0.128	1.053	−0.122	0.015	0.903	0.879 (0.112–6.920)
SAA	0.012	0.005	2.511	6.304	0.012	1.012 (1.003–1.022)
ΔHb	0.13	0.042	3.106	9.644	0.002	1.139 (1.049–1.237)
Asphyxia	2.941	1.849	1.591	2.531	0.112	18.938 (0.506–709.362)
Ventilator usage	−2.386	1.598	−1.493	2.229	0.135	0.092 (0.004–2.110)

GA, gestational age; BW, birth weight; WBC, white blood cell count; N, neutrophil count; L, lymphocyte count; SAA, serum amyloid A; ΔHb, hemoglobin decline.

### ROC curve analysis for NEC prediction in LOS

**Figure 2:**
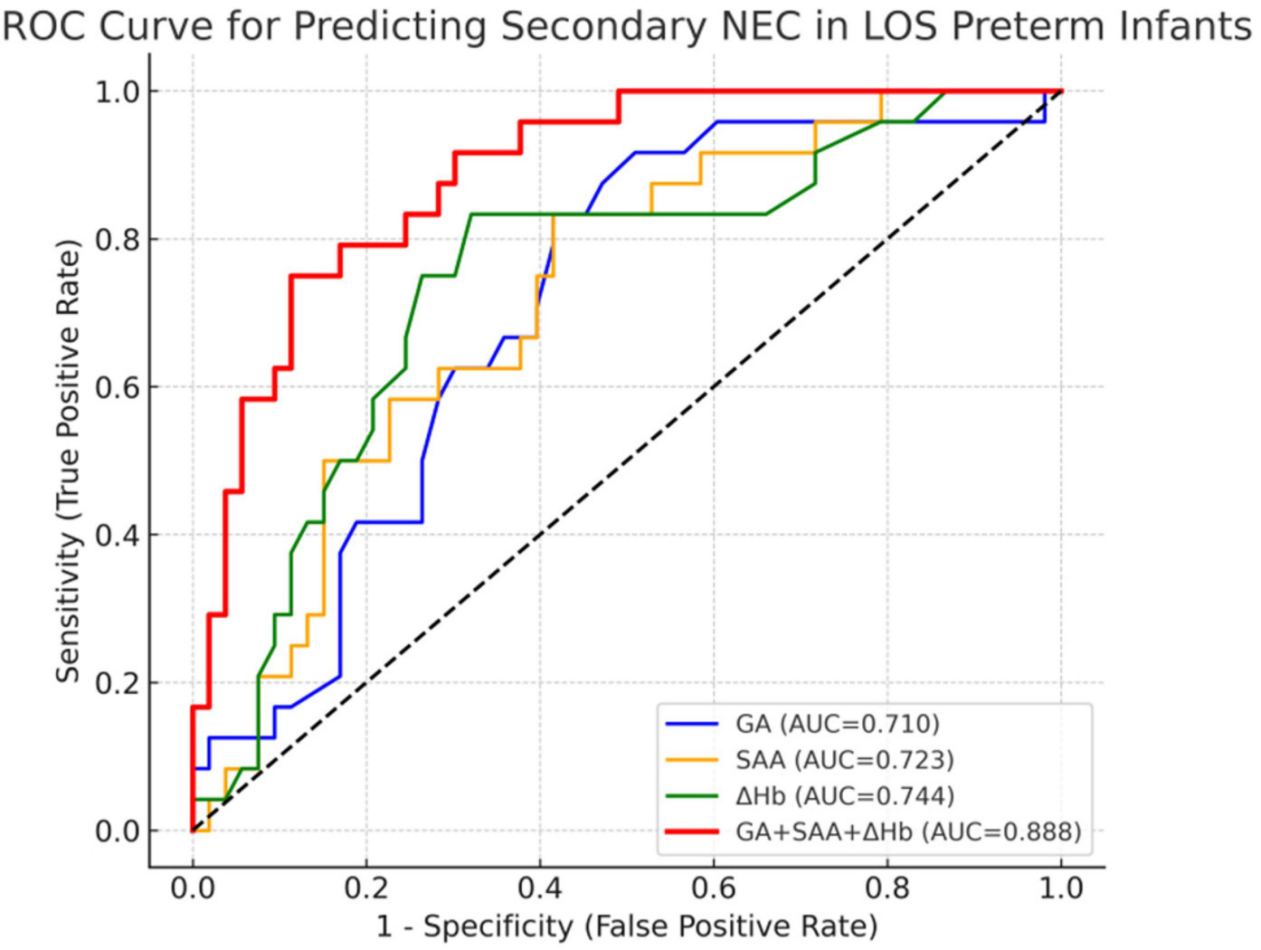
The ROC curve of gestational age, serum SAA and ΔHb combined to predict the secondary NEC of LOS in premature infants.

**Figure 3:**
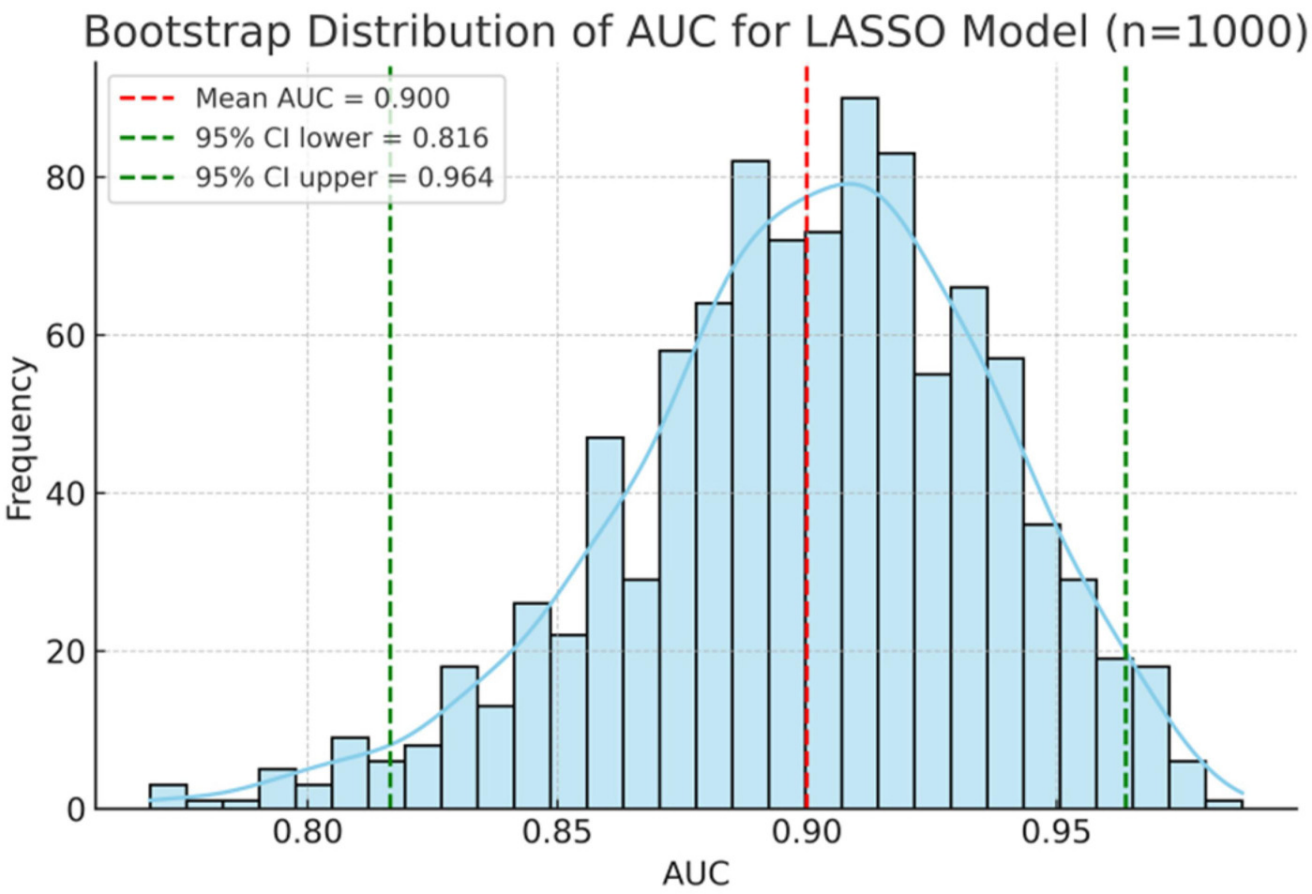
Predictive performance of biomarkers and the LASSO model for NEC econdary to LOS. Bootstrap distribution of AUC for the LASSO model including GA, SAA, and ΔHb. The histogram and density curve (*n* = 1,000 resamples) demonstrate a mean AUC of 0.900 with 95% CI of 0.816–0.964.

**Figure 4:**
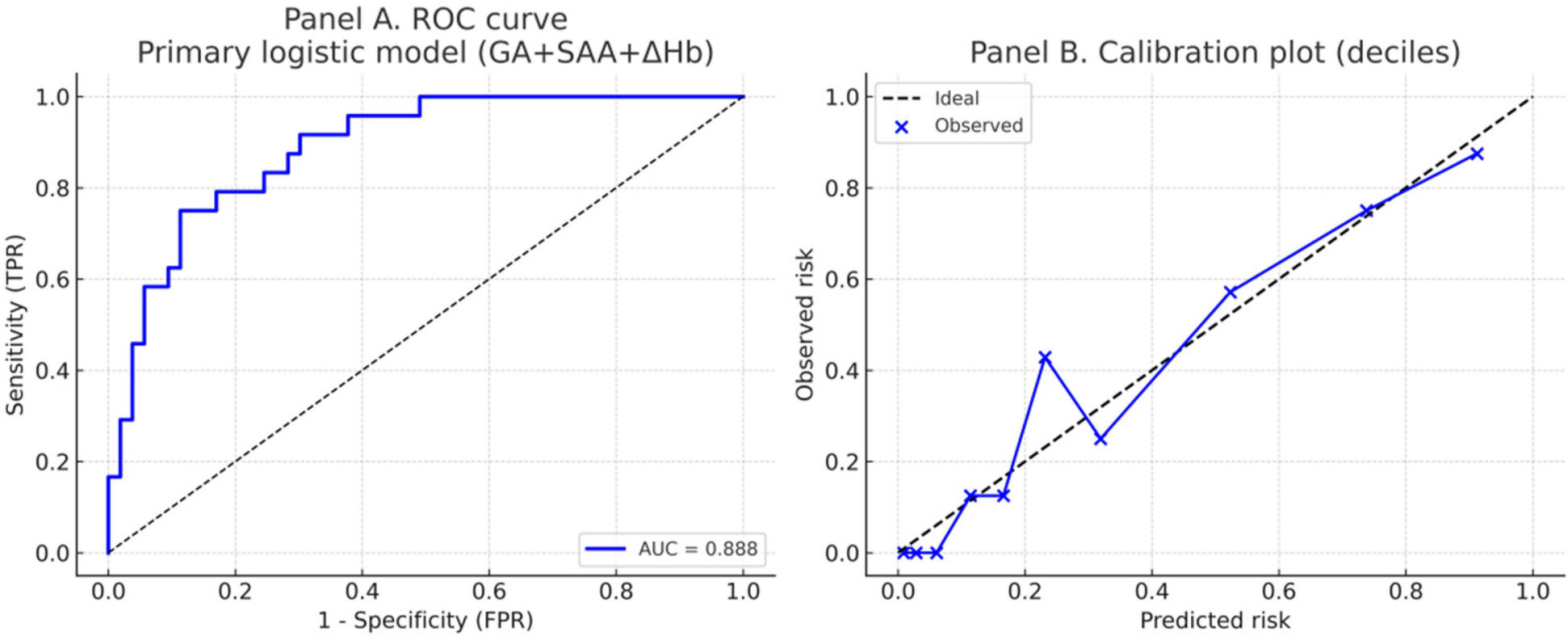
Discrimination and calibration of the primary logistic regression model predicting NEC secondary to LOS. Panel **(A)** Receiver operating characteristic (ROC) curve of the primary model including gestational age (GA), serum amyloid A (SAA), and hemoglobin decline (ΔHb). The model achieved an AUC of 0.888. Panel **(B)**. Calibration plot using deciles of predicted risk. The dashed line indicates perfect calibration, while blue points and the connecting line represent observed vs. predicted probabilities. The model demonstrated good calibration (intercept = 0.001, slope = 1.001; Brier score = 0.126).

As shown in Figure 2; Table 4, ROC analysis showed that the optimal cut-off values for predicting NEC in LOS preterm infants were 236 days for gestational age, 78.3 mg/L for SAA, and 15 g/L for ΔHb. In the ROC analysis, gestational age (GA), serum amyloid A (SAA), and hemoglobin decline (ΔHb) showed moderate predictive ability for NEC secondary to LOS, with AUC values of 0.710, 0.723, and 0.746, respectively. The combined model of GA + SAA+ΔHb achieved the highest predictive accuracy (AUC = 0.888, 95% CI: 0.810–0.953). To further enhance model robustness, we applied LASSO regression for variable selection, which retained all three predictors: GA (days), SAA (mg/L), and ΔHb (g/L). As shown in Figures 3, 4, internal validation via 1,000 bootstrap resamples demonstrated high stability of the LASSO model, yielding a mean AUC of 0.900 (SE = 0.037, 95% CI: 0.816–0.964). Calibration performance was excellent (intercept = 0.001; slope = 1.001), and the Brier score was 0.126. These results indicated that the integrated LASSO model provides a superior and reliable predictive tool compared to any single biomarker. Furthermore, the primary (unpenalized) logistic regression model including the same three variables achieved an apparent AUROC of 0.888 and a bootstrap optimism-corrected AUROC of 0.872 (*n* = 1,000), further supporting the model's robustness.

**Table 4:** ROC curve analysis of GA, SAA and ΔHb in predicting secondary NEC in LOS preterm infants.

Item	AUC	p	95% CI	Sensitivity	Specificity	Youden's index	Cut-off	PPV	NPV
GA	0.710	0.002	0.584–0.819	0.833	0.585	0.418	236	0.476	0.886
SAA	0.723	0.001	0.617–0.836	0.833	0.585	0.418	78.3	0.476	0.886
ΔHb	0.744	0.001	0.616–0.860	0.833	0.679	0.513	15	0.541	0.900
SAA+*Δ*Hb	0.792	0.000	0.690–0.888	0.750	0.774	0.524	0.302	0.302	0.872
GA + SAA+ΔHb	0.888	0.000	0.810–0.953	0.750	0.887	0.637	0.401	0.401	0.887

GA, gestational age; BW, birth weight; SAA, serum amyloid A; ΔHb, hemoglobin decline.

## Discussion

Due to the immature immune system in neonates, sepsis often progresses rapidly from subclinical symptoms to severe systemic infections, which can damage multiple organs and lead to various complications, with NEC being the most common. NEC significantly increases the mortality rate among preterm infants. Studies have indicated that sepsis is a risk factor for NEC (5). The clinical presentation of LOS in preterm infants is often atypical, leading to misdiagnosis or delayed diagnosis and inappropriate antibiotic use. Therefore, exploring predictive indicators for NEC secondary to LOS in preterm infants and implementing early interventions are crucial for improving outcomes and reducing mortality.

Guided by the aforementioned pathophysiology, we pre-specified three key variables for investigation: gestational age (GA) as a marker of intrinsic vulnerability, serum amyloid A (SAA) as a sensitive indicator of the systemic inflammatory burden, and hemoglobin decline (ΔHb) as a potential marker of the hematological sequelae of severe infection. Our objective was not to discover novel markers through an unbiased search but to validate the combined utility of this biologically plausible set of parameters for risk stratification in a defined clinical scenario (i.e., LOS).

### Impact of gestational age on NEC secondary to LOS in preterm infants

This study found that gestational age was significantly lower in the NEC group compared to the non-NEC group. Logistic regression analysis further confirmed that lower gestational age was a risk factor for developing NEC secondary to LOS, which aligns with previous research (15, 16).

### Effect of SAA on NEC secondary to LOS in preterm infants

SAA is a precursor protein involved in inflammation-related amyloidosis. It is induced by IL-1, IL-6, TNF-α, and Gram-negative bacterial lipopolysaccharides, and can increase significantly in the early stages of systemic infections. SAA is highly sensitive and can be monitored over a prolonged period, making it a promising biomarker for diagnosing and managing neonatal sepsis (17). A meta-analysis of nine studies involving 823 neonates showed that SAA has high diagnostic value at 0 h and 8–96 h after the onset of sepsis symptoms. In general, SAA demonstrated greater diagnostic accuracy than CRP, with a combined sensitivity of 0.84 and specificity of 0.89 at 0 h (18). An additional advantage of SAA is that it does not require additional blood sampling, which reduces iatrogenic blood loss and may be more suitable for preterm infants. In premature infants, Arnon et al. (19) found that SAA levels were elevated in the LOS group, and this increase was more pronounced in cases of gram-negative bacterial LOS. Compared to CRP and WBC, SAA exhibited the highest sensitivity (100%), specificity (93%), and positive predictive value (PPV, 96%). This study found that SAA levels were significantly higher in the NEC group compared to the non-NEC group, and logistic regression analysis indicated that elevated SAA was a risk factor for NEC secondary to LOS.

### Effect of ΔHb on NEC secondary to LOS in preterm infants

Anemia may be associated with adverse outcomes in preterm infants with infectious shock (20). Moreover, infants with sepsis who also have anemia, particularly moderate or severe anemia, have a higher risk of developing NEC compared to those with mild or no anemia (21). During infection, Hb levels often decline, resulting in true or relative anemia. Possible mechanisms include reduced erythropoiesis due to systemic inflammation, shortened erythrocyte lifespan, and increased destruction, as well as dilutional effects from intravenous fluids. However, there are few studies assessing the severity of sepsis and risk of secondary NEC based on ΔHb. This study found no significant difference in Hb levels between the NEC and non-NEC groups before or at the onset of LOS, but the NEC group exhibited significantly greater ΔHb. Logistic regression analysis identified ΔHb as an independent risk factor for NEC secondary to LOS.

Further ROC analysis in this study suggested that the optimal thresholds for predicting NEC secondary to LOS in preterm infants were 236 days for gestational age, 78.3 mg/L for SAA, and 15 g/L for ΔHb. The combination of these three indicators yielded a Youden index of 0.637, an area under the curve of 0.888, a sensitivity of 0.750, and a specificity of 0.887, demonstrating improved predictive accuracy when used together. The high Negative Predictive Value (NPV = 0.887) means a negative result is very useful for “ruling out” imminent NEC, potentially allowing for less aggressive management in low-risk infants and optimizing resource allocation. The Positive Predictive Value (PPV = 0.401) indicates that a positive result identifies a group at substantially higher risk than the baseline prevalence. This would justify targeting high-risk infants for more intensive monitoring (e.g., more frequent abdominal exams, delayed feeding advancement), which could lead to earlier detection and intervention.

### Limitations

This study had several limitations that must be acknowledged. First, its retrospective, single-center design inherently introduces potential selection and information biases. The relatively small sample size, particularly the number of NEC events (*n* = 24), limits the statistical power and the generalizability of our findings. Although we employed bootstrap internal validation to obtain a more robust estimate, future studies with larger cohorts are necessary for confirmation.

Second, the timing of SAA measurement was defined as within 24 h of clinical onset but may not have captured the peak level for every infant, as SAA kinetics can vary based on the pathogen and host response. Furthermore, due to the low rate of pathogen identification in clinical practice and our cohort, we were unable to perform a stratified analysis based on microbial etiology (e.g., gram-positive vs. gram-negative bacteria vs. viruses). The predictive value of SAA and the proposed cut-off value might differ according to the causative pathogen; thus, our results should be interpreted as foundational, and pathogen-specific thresholds should be established in future research.

Third, due to the retrospective design, we could not calculate the rate of hemoglobin decline (ΔHb per day) or adjust for the precise volume of IV fluids administered, which might contribute to hemodilution. Future prospective studies should record exact phlebotomy times and fluid balances to confirm our findings.

Furthermore, due to the retrospective design and limited sample size, we cannot rule out the influence of unmeasured temporal changes in practice, nor assess the model's stability over time. The limited number of events also affects the precision of our estimates, as seen in the ROC curve. Although bootstrap validation was used to provide a more generalizable performance estimate, future prospective multi-center studies are needed to confirm the temporal validity and robustness of our findings.

Fourth, a key limitation is our inability to analyze the data based on the pathogenic cause of LOS. The host response, including SAA kinetics and the degree of hemoglobin decline, likely differs between bacterial, viral, and fungal infections. Unfortunately, the low rate of pathogen identification in our cohort precluded any meaningful stratified analysis. Therefore, our findings and the proposed cut-off values represent a composite risk profile across all LOS types. They should be interpreted as a proof-of-concept that these markers have utility in the initial risk assessment before an etiology is established. Future large-scale, multi-center studies with systematic molecular testing are urgently needed to validate our model and to investigate pathogen-specific risk algorithms.

## Clinical implications and future directions

Despite these limitations, the combination of GA, SAA, and ΔHb offers a pragmatic approach for early risk stratification in a clinical setting where decisions must be made rapidly, often before pathogen identification. These biomarkers are routinely measured in the standard workup of late-onset sepsis, making our model highly feasible and cost-effective to implement immediately.

We envision a clinical pathway where upon suspicion of LOS, a preterm infant's risk of progressing to NEC can be quickly assessed using these three parameters. Infants identified as high-risk (e.g., those meeting the cut-off values of GA < 236 days, SAA > 78.3 mg/L, and ΔHb > 15 g/L) could be managed with a higher level of vigilance. This might include more frequent abdominal examinations, delayed or more cautious advancement of enteral feeds, and a lower threshold for obtaining diagnostic imaging. Conversely, this model could also help identify a lower-risk group in whom overly aggressive management might be avoided.

Ultimately, the goal is to move towards personalized medicine in NEC prevention. Before this model can be formally integrated into clinical guidelines, its utility in actually improving patient outcomes must be validated in large, prospective, multi-center interventional studies. Such trials could randomize high-risk infants identified by the model to different monitoring or preventive strategies (e.g., stricter feeding protocols, potential future pharmacologic agents) to determine if early risk stratification can effectively reduce NEC incidence and mortality.

## Conclusion

Gestational age, SAA, and ΔHb are important markers for assessing the risk of NEC secondary to LOS in preterm infants. Higher SAA and ΔHb, along with lower gestational age, indicate a higher likelihood of NEC development. The combination of these markers provides a potentially convenient, cost-effective, and minimally invasive method for the early risk stratification of NEC secondary to LOS. However, given the retrospective nature and limitations discussed above, our findings are preliminary and must be validated in larger, multi-center prospective studies before they can be considered for clinical application.

## Data Availability

The original contributions presented in the study are included in the article/Supplementary Material, further inquiries can be directed to the corresponding author.
