# Supplementary material for: Evaluation of gestational age, serum amyloid A, and hemoglobin decline (ΔHb) as diagnostic markers for NEC secondary to late-onset sepsis in preterm infants
Source: Front Pediatr. 2025 Oct 9;13:1662371. doi: 10.3389/fped.2025.1662371 (PMC12545128; doi:10.3389/fped.2025.1662371)
Supplement: Supplementary file 1 [file Supplementaryfile1.docx]

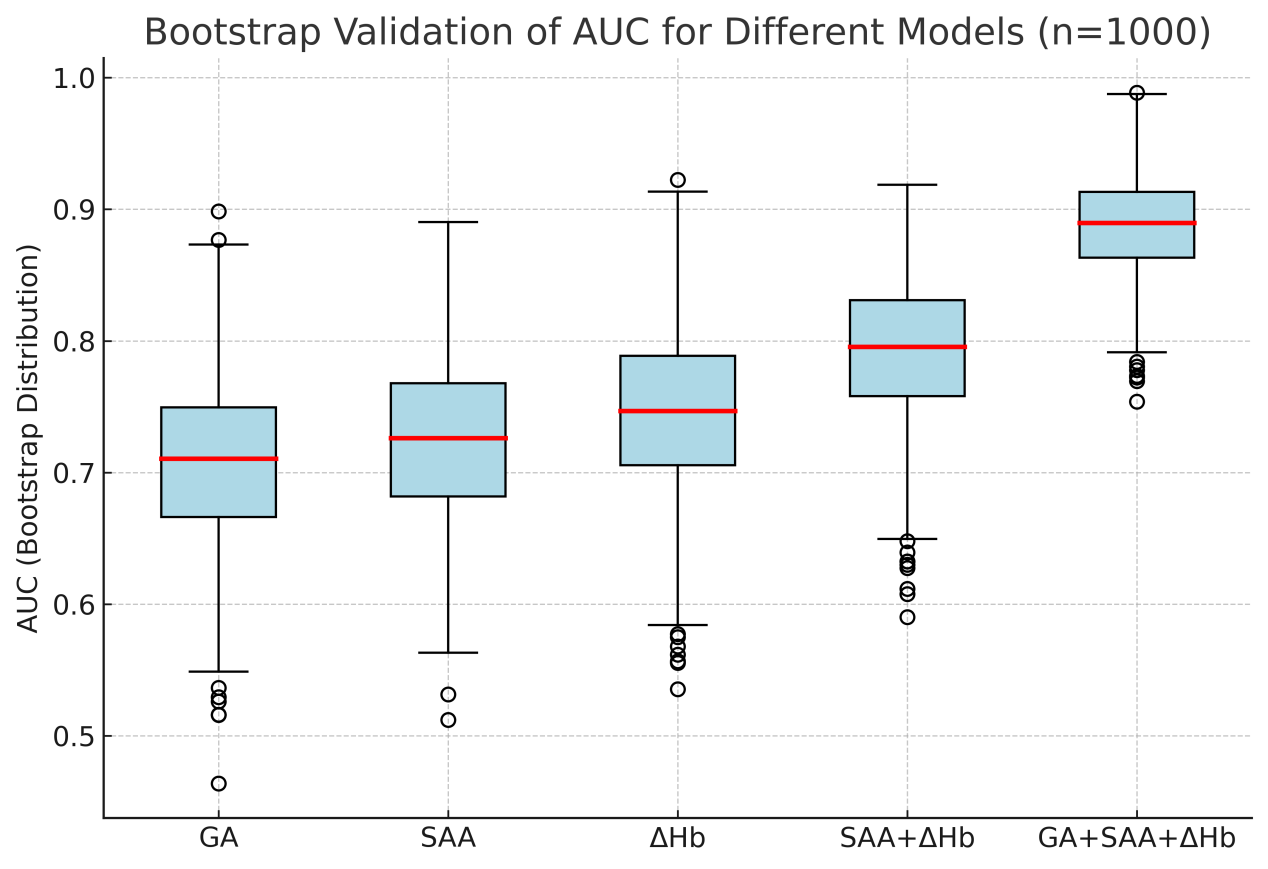


Supplementary Figure S1. Bootstrap validation of single predictors and simple combinations. Boxplots illustrate the distribution of AUC values across 1000 resamples for GA, SAA, ΔHb, and SAA+ΔHb, showing limited stability compared with the integrated LASSO model.
